# PCA-Based Multiple-Trait GWAS Analysis: A Powerful Model for Exploring Pleiotropy

**DOI:** 10.3390/ani8120239

**Published:** 2018-12-17

**Authors:** Wengang Zhang, Xue Gao, Xinping Shi, Bo Zhu, Zezhao Wang, Huijiang Gao, Lingyang Xu, Lupei Zhang, Junya Li, Yan Chen

**Affiliations:** 1 Cattle Genetics and Breeding Group, Institute of Animal Science (IAS), Chinese Academy of Agricultural Sciences (CAAS), Beijing 100193, China; zhangwengang_19@sina.com (W.Z.); gaoxue76@126.com (X.G.); sxp18811727129@163.com (X.S.); zhubo525@126.com (B.Z.); wangzezhao1@163.com (Z.W.); gaohj111@sina.com (H.G.); xulingyang@caas.cn (L.X.); zhanglupei@caas.cn (L.Z.); 2 College of Animal Science and Technology, Hebei Agricultural University, Baoding 071000, China

**Keywords:** genome-wide association study, principal component analysis, multiple-trait, pleiotropy, *MCHR2*

## Abstract

**Simple Summary:**

In biological processes, it is common that a single gene controls two or more traits, leading to a high genetically correlation between many traits in human beings and livestock. Genome-wide association study (GWAS) is a popular method for mapping causal genes or regions related to studied traits. Taking the advantage of genetically correlation among traits, a combined analysis of two or more traits can improve the power of detection in GWAS analysis. In this study, we prove the improvement of multiple-traits GWAS through theoretical derivation, simulated dataset and real dataset, respectively. In addition, using this approach, we successfully identified a candidate gene for presoma muscle development in cattle that were not be found in the average association analysis. In summary, we conclude that multiple-trait GWAS is an effective method to explore genetic factors of traits, which have high correlations.

**Abstract:**

Principal component analysis (PCA) is a potential approach that can be applied in multiple-trait genome-wide association studies (GWAS) to explore pleiotropy, as well as increase the power of quantitative trait loci (QTL) detection. In this study, the relationship of test single nucleotide polymorphisms (SNPs) was determined between single-trait GWAS and PCA-based GWAS. We found that the estimated pleiotropic quantitative trait nucleotides (QTNs) $\hat{\beta^{*}}$ were in most cases larger than the single-trait model estimations ($\hat{\beta_{1}}$ and $\hat{\beta_{2}}$). Analysis using the simulated data showed that PCA-based multiple-trait GWAS has improved statistical power for detecting QTL compared to single-trait GWAS. For the minor allele frequency (MAF), when the MAF of QTNs was greater than 0.2, the PCA-based model had a significant advantage in detecting the pleiotropic QTNs, but when its MAF was reduced from 0.2 to 0, the advantage began to disappear. In addition, as the linkage disequilibrium (LD) of the pleiotropic QTNs decreased, its detection ability declined in the co-localization effect model. Furthermore, on the real data of 1141 Simmental cattle, we applied the PCA model to the multiple-trait GWAS analysis and identified a QTL that was consistent with a candidate gene, *MCHR2*, which was associated with presoma muscle development in cattle. In summary, PCA-based multiple-trait GWAS is an efficient model for exploring pleiotropic QTNs in quantitative traits.

## 1. Introduction

Disease and quantitative traits usually follow a polygenic model [1], in which quantitative trait loci (QTL) and candidate genes can be explored using genome-wide association studies (GWAS) [2]. In general, candidate genes or causal variants can affect multiple traits simultaneously, a phenomenon known as “pleiotropy”, that usually occurs when traits share common quantitative trait nucleotides (QTNs), or QTNs in traits have a high linkage disequilibrium (LD) [3]. Typical pleiotropic traits are phenotypically or genetically correlated and are unconstrained, such as disease traits, quantitative traits, and Mendelian traits. According to the National Human Genome Research Institute (NHGRI) [4], pleiotropy exists in 17% of trait-associated genes and 5% of trait-associated single nucleotide polymorphisms (SNPs). Studies on Crohn’s disease and psoriasis [5], and body mass index (BMI) and melanoma [6], have highlighted numerous pleiotropic QTNs.

A plausible approach for exploring pleiotropy is the multiple-trait GWAS model in comparison with single trait GWAS, which has been shown to be an effective method to detect shared QTL [7]. Although a multivariate model with multiple traits is a powerful approach, it requires a large amount of computation time and computational memory capacity [8], because it must solve a covariance matrix of *np* × *np* in size (*n*, number of individuals; *p*, number of traits), with a time complexity of O(*n*^3^*p*^3^·*t*). Some researchers [9,10,11] have reduced the computation time, however the multivariate model is still costly when many traits are considered together. Based on principal component analysis (PCA) and linear discriminant analysis, another powerful model utilizes dimension reduction of traits to track pleiotropy [12,13]. PCA-based multiple-trait GWAS has been shown to explain the largest amount of heritability [14], as well as to be robust and powerful in practice [15]. Compared with the multivariate model, this method takes much less time, therefore it has been widely used in pleiotropic QTL mapping [16]. However, it should be noted that one limitation of PCA-based GWAS is that it can only be applied when all traits are measured on all samples.

In livestock breeding, fine mapping of pleiotropic QTL for objective traits, such as milk yield, milk fat yield, and milk protein yield in dairy cattle [17,18], as well as the average daily gain and carcass weight in beef cattle [19], is important. Christine conducted a PCA-based multiple-trait GWAS and identified two regions (SSC5: 21.3 Mb–25.1 Mb, SSC14: 151.5 Mb–154.0 Mb) that have pleiotropic effects on boar taint components and testicular traits [20]. It helps to better understand the genetic mechanisms of complex traits, especially those related to commercial traits, and provide guidance for marker-assisted selection (MAS) in domestic animal breeding.

**Figure 1:**
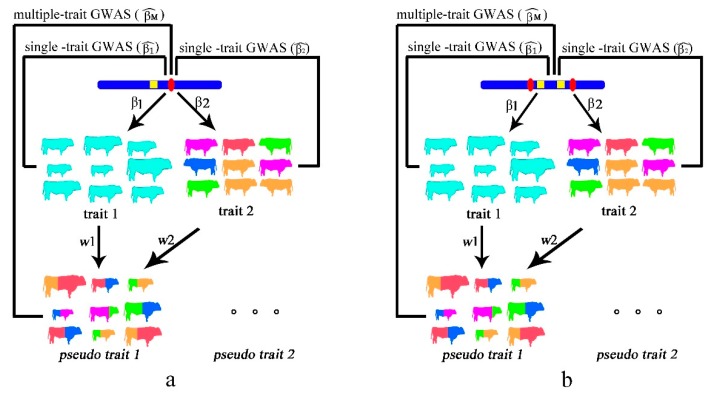
Layout of principal component analysis (PCA)-based multiple-trait genome-wide association studies (GWAS) versus single-trait GWAS. (**a**) Single causal variant model. Provided that a casual single nucleotide polymorphism (SNP) (red spot) has an effect on trait 1 (cattle size) and trait 2 (cattle color) with *β*1 and *β*2, the process of estimation of *β*1 and *β*2 using trait 1 and trait 2 is called single-trait GWAS. According to components decomposition, pseudo traits are formed and the process of estimation of *β_M_* is called PCA-based multiple-trait GWAS. The yellow marker represents genotyped SNP in beadchip. (**b**) Colocalizing effect model. Two different genetic variants in high linkage disequilibrium that affect different traits. In both situations, we compared the relationships among *β*1, *β*2, and *β_M_*.

In this study, we considered two types of pleiotropy, namely a single causal variant model and a colocalizing effect model. Specifically, the colocalizing effect model is defined as different causal variants that affect distinguishing phenotypes with high linkage disequilibrium (LD), resulting in variants displaying signals in association with different traits. We first theoretically describe the relationship between a PCA-based multiple-trait GWAS model and single-trait model for pleiotropic QTL mapping. Next, we demonstrate a powerful PCA-based model based on three sets of simulation data under three situations (medium heritability traits, low heritability traits, and environmental correlation traits). Finally, we use real GWAS data of three meat cut traits to explore candidate genes associated with presoma development in cattle. The analytical strategies are visually outlined in Figure 1.

## 2. Method

We firstly decomposed the phenotypes into several principal components scores (PCS) according to eigenvectors, and then treated PCS as pseudo traits to carry out multiple-trait GWAS. To show the improved power of PCA-based GWAS, we theoretically explored the relationship of the estimated effects between PCA-based multiple-trait GWAS and single-trait GWAS. In this study, two situations were considered as follows.

### 2.1. Single Causal Variant Model

In GWAS analysis, the standard approach usually uses a mixed linear model (MLM), in which polygenic effects are treated as random effects [21]. For a clearer comparison with the two association strategies (multi-traits GWAS and single-trait GWAS), we simplified the GWAS model into a general linear model (GLM) instead of a MLM (Figure 1). Here, we referred to a GLM in a QTL mapping study [22] (also called least-squares regression if only a SNP effect is considered in the model). *X* is the genotype matrix for a single marker, defined as 0 for the heterozygote and −1 and 1 for the two homozygotes. Two traits were observed (represented by *y*_1_ and *y*_2_) and included in single-marker GLM tests as follows:

(1) $$y_{1}=X\beta_{1}+e_{1}$$

(2) $$y_{2}=X\beta_{2}+e_{2}$$

where *β*_1_ and *β*_2_ represent the marker’s effect on trait one and trait two, respectively. Therefore, *β*_1_ and *β*_2_ are estimated by

(3) $$\hat{\beta_{1}}={\left(X^{T}X\right)}^{-1}X^{T}y_{1}$$

(4) $$\hat{\beta_{2}}={\left(X^{T}X\right)}^{-1}X^{T}y_{2}$$

The phenotypes followed E(*y*_1_) = 0 and E(*y*_2_) = 0 after phenotype normalization. We conducted principal component analysis (PCA) between phenotypic traits in two steps. First, we constructed the covariance matrix *S*:

(5) $$S=\left[\begin{array}{cc} \frac{{\left(y_{1}-{\bar{y}}_{1}\right)}^{T}\left(y_{1}-{\bar{y}}_{1}\right)}{n-1} & \frac{{\left(y_{1}-{\bar{y}}_{1}\right)}^{T}\left(y_{2}-{\bar{y}}_{2}\right)}{n-1}\\ \frac{{\left(y_{2}-{\bar{y}}_{2}\right)}^{T}\left(y_{1}-{\bar{y}}_{1}\right)}{n-1} & \frac{{\left(y_{2}-{\bar{y}}_{2}\right)}^{T}\left(y_{2}-{\bar{y}}_{2}\right)}{n-1} \end{array}\right]=\frac{1}{n-1}\left[\begin{array}{cc} y_{1}^{T}y_{1} & y_{1}^{T}y_{2}\\ y_{2}^{T}y_{1} & y_{2}^{T}y_{2} \end{array}\right]$$

where *n* is the number of phenotyped individuals. Second, we created a pseudo trait weighting of the first eigenvector (*μ*):

(6) $$y^{*}=\left[y_{1},y_{2}\right]\mu$$

Therefore, the linear regression analysis and marker’s effect estimation of β* can be written as

(7) $$y^{*}=X\beta^{*}+e^{*}$$

(8) $$\hat{\beta^{*}}={\left(X^{T}X\right)}^{-1}X^{T}y^{*}$$

Here, we compared the pseudo trait effect (β*) with two traits effects (*β*_1_ and *β*_2_) to explain the increasing power using the pseudo trait. Since

(9) $${\left(\hat{\beta_{2}}\right)}^{T}\hat{\beta_{1}}=y_{2}^{T}X{\left(X^{T}X\right)}^{-1}{\left(X^{T}X\right)}^{-1}X^{T}y_{1}={\left(X^{T}X\right)}^{-2}y_{2}^{T}XX^{T}y_{1}$$

(10) $${\left(\hat{\beta_{1}}\right)}^{T}\hat{\beta_{2}}=y_{1}^{T}X{\left(X^{T}X\right)}^{-1}{\left(X^{T}X\right)}^{-1}X^{T}y_{2}={\left(X^{T}X\right)}^{-2}y_{1}^{T}XX^{T}y_{2}$$

(11) $${\left(\hat{\beta_{1}}\right)}^{T}=\hat{\beta_{1}};\;{\left(\hat{\beta_{1}}\right)}^{T}=\hat{\beta_{1}}$$

we had

(12) $$\hat{\beta_{1}}\hat{\beta_{2}}{\left(X^{T}X\right)}^{2}=y_{2}^{T}XX^{T}y_{1}<ny_{2}^{T}y_{1}$$

Putting Equation (12) into Equation (5) we got

(13) $$S>\frac{{\left(X^{T}X\right)}^{2}}{n\left(n-1\right)}\left[\begin{array}{cc} \beta_{1}\beta_{1} & \beta_{1}\beta_{2}\\ \beta_{2}\beta_{1} & \beta_{2}\beta_{2} \end{array}\right]$$

Because Sμ=λμ, where *λ* was the eigenvalue corresponding to *μ*, we had

(14) $$\lambda \hat{\beta^{*}}={\left(X^{T}X\right)}^{-1}X^{T}\left[y_{1},y_{2}\right]\lambda \mu ={\left(X^{T}X\right)}^{-1}X^{T}\left[y_{1},y_{2}\right]S\mu$$

Putting Equation (13) into Equation (5) we got

(15) $$\begin{array}{l} \lambda \hat{\beta^{*}}>\frac{{\left(X^{T}X\right)}^{2}}{n\left(n-1\right)}{\left(X^{T}X\right)}^{-1}X^{T}\left[y_{1},y_{2}\right]\left[\begin{array}{cc} \beta_{1}\beta_{1} & \beta_{1}\beta_{2}\\ \beta_{2}\beta_{1} & \beta_{2}\beta_{2} \end{array}\right]\mu \\ \;\;\;\;\;\;\;=\frac{{\left(X^{T}X\right)}^{2}}{n\left(n-1\right)}{\left(X^{T}X\right)}^{-1}X^{T}\left[X\hat{\beta_{1}}+e_{1},X\hat{\beta_{2}}+e_{2}\right]\left[\begin{array}{cc} \beta_{1}\beta_{1} & \beta_{1}\beta_{2}\\ \beta_{2}\beta_{1} & \beta_{2}\beta_{2} \end{array}\right]\mu \end{array}$$

By letting $B=\left[\hat{\beta_{1}},\hat{\beta_{2}}\right]$ and inserting *λ* into right-hand side, we got

(16) $$\hat{\beta^{*}}>\mu \frac{X^{T}\left[X,1\right]\left[\begin{array}{cc} \beta_{1} & \beta_{2}\\ e_{1} & e_{2} \end{array}\right]\left[\begin{array}{c} \beta_{1}\\ \beta_{2} \end{array}\right]\left[\beta_{1},\beta_{2}\right]\left(X^{T}X\right)}{n\left(n-1\right)\lambda }=\frac{X^{T}XBB^{T}B\mu \left(X^{T}X\right)}{n\left(n-1\right)\lambda }+\frac{X^{T}e_{1}\beta_{1}B\mu \left(X^{T}X\right)}{n\left(n-1\right)\lambda }+\frac{X^{T}e_{2}\beta_{2}B\mu \left(X^{T}X\right)}{n\left(n-1\right)\lambda }$$

The residual error can be considered to be independent of the marker indicator matrix *X*. $E\left(e_{1}\right)=0$ results in $E\left(\frac{X^{T}e_{1}\beta_{1}B\mu \left(X^{T}X\right)}{n\left(n-1\right)\lambda }\right)=0$ and $E\left(\frac{X^{T}e_{2}\beta_{2}B\mu \left(X^{T}X\right)}{n\left(n-1\right)\lambda }\right)=0$. Provided that the phenotypic correlation coefficient approaches 1, the first eigenvalue can be considered to be

(17) $$\lambda \overset{cor\left(y_{1},y_{2}\right)\to 1}{\to }trS=\frac{\left(\beta_{1}^{2}+\beta_{2}^{2}\right)\left(X^{T}X\right)}{n\left(n-1\right)}.$$

Therefore, putting Equation (17) into Equation (16), we obtained the β* estimation:

(18) $$\hat{\beta^{*}}>\frac{X^{T}XBB^{T}B\mu \left(X^{T}X\right)}{n\left(n-1\right)\lambda }=\frac{BB^{T}B\mu }{\beta_{1}^{2}+\beta_{2}^{2}}=B\mu =\hat{\beta_{1}}w_{1}+\hat{\beta_{2}}w_{2}$$

where *w*_1_ and *w*_2_ represent elements of the eigenvector *μ*.

For pleiotropic SNPs, this result indicated that the PCA-based multiple-trait model had a high chi-square statistic for the tested SNP compared to the single-trait model.

### 2.2. Colocalizing Effect Model

As shown in Figure 1, we assumed that marker 1 had a genuine effect on trait 1, marker 2 had a genuine effect on trait 2, and both were located in the same gene, or within a short distance with a strong linkage disequilibrium (LD). The LD level of the two markers was $r_{LD}=\frac{1}{n}{X_{1}}^{T}X_{2}$, where *X*_1_ and *X*_2_ are the normalized genotypes, with E(*X*_1_) = E(*X*_2_) = 0 and Var (*X*_1_) = Var (*X*_2_) = 1. Similarly, the effects of marker 1 on trait one, marker 2 on trait two, and marker 1 on a pseudo trait are *β*_1_, *β*_2_, and β*, respectively, as in Equations (3)–(5).

Since

(19) $${\left(\hat{\beta_{2}}\right)}^{T}\hat{\beta_{1}}=y_{2}^{T}X_{2}{\left({X_{2}}^{T}X_{2}\right)}^{-1}{\left({X_{1}}^{T}X_{1}\right)}^{-1}{X_{1}}^{T}y_{1}=n^{-2}r^{-1}y_{2}^{T}y_{1}$$

we had

(20) $$\hat{\beta_{1}}\hat{\beta_{2}}nr<{y_{1}}^{T}y_{2}$$

(21) $$S>\frac{nr}{n-1}\left[\begin{array}{cc} y_{1}^{T}y_{1} & y_{1}^{T}y_{2}\\ y_{2}^{T}y_{1} & y_{2}^{T}y_{2} \end{array}\right]$$

Next, we performed a derivation to estimate β* as in the *single causal variant model*—Equations (13)–(16). Therefore, we had

(22) $$\hat{\beta^{*}}>\frac{n^{2}rBB^{T}B\mu }{\left(n-1\right)\lambda }=r\left(\hat{\beta_{1}}w_{1}+\hat{\beta_{2}}w_{2}\right)$$

### 2.3. Simulated Data

We simulated phenotypes based on real data that included 1000 samples and 120,710 SNPs on five chromosomes. The principle of phenotypic simulation is as follows:

$$y=\sum_{i}X_{i}\alpha_{i}+g+\varepsilon$$

where $g\sim N\left(0,G\sigma_{g}^{2}\right)$ for which $\sigma_{g}^{2}$ is the additive genetic variance and G is the genomic relationship matrix. *α*_i_ is the *i*th quantitative trait nucleotide (QTN) effect followed by a gamma distribution with a shape parameter of 0.4 and scale parameter 1.66. The polygenic effects vector g was formed by $g={\left(G^{\frac{1}{2}}\sigma_{g}\right)}^{T}\tau$, with τ following a normal distribution. The total additive genetic variance can be written as $\sigma_{T}^{2}=\sum \sigma_{i}^{2}+\sigma_{g}^{2}$, and the residual error as $\varepsilon \sim N\left(0,\;\frac{\left(1-h^{2}\right)\sigma_{T}^{2}}{h^{2}}\right)$. For the pleiotropic traits simulations, we assumed that each two traits shared 10 common QTNs that contributed 50% of the total genetic variance ($\sigma_{T}^{2}$).

When simulating low heritability traits, we set the parameters as *h*_2_ = 0.05 and *r*(*e*_1_,*e*_2_) = 0. When simulating environmental correlation traits, we set the parameters as *h*_2_ = 0.5 and *r*(*e*_1_,*e*_2_) = 0.25.

### 2.4. Real Data

In the GWAS analysis, a total of 1141 Simmental beef cattle born between 2008 and 2014 composed the experimental population. All cattle were from more than 30 families and were fattened for 8–12 months in a similar environment with the same feed, and slaughtered following the Standard Wholesale Cuts of American Beef guidelines. The phenotypes of three meat cut traits, including the clod weight (CW), fore shank weight (FSW), and heel muscle shank weight (HMSW), were collected during slaughtering. DNA was extracted from the blood samples and genotyped using an Illumina BovineHD BeadChip (Illumina, CA, USA).

Quality control was conducted as follows: (1) Individuals with a call rate < 0.95 and SNPs with a call rate < 0.9 were removed, (2) minor allele frequency < 0.05, and (3) *p*-Value of Hardy–Weinberg equilibrium < 10^−6^. Finally, a total of 1111 individuals and 608,761 SNPs were left for subsequent analysis. In this study, all phenotypes followed normal distribution and GWAS analyses were implemented using a mixed linear model (MLM). PCA was performed by SAS (Statistical Analysis System) software version 9.4 (SAS Institute Inc., Cary, NC, USA) and genetic parameter estimations were conducted using GCTA (Genome-wide Complex Trait Analysis) [23].

### 2.5. Power Examination and False Discovery Rate (FDR) Examination

Based on the simulated phenotypes, the power and FDR were calculated under different significant thresholds using a single-trait model and PCA-based multiple-trait model. Power was evaluated as the proportion of QTNs that passed the significance threshold. FDR was defined as the proportion of the non-QTN markers among the identified markers that exceeded the threshold, where the non-QTN markers were markers that were not located 10 Kb upstream or downstream of the QTNs. A total of 100 replicates were conducted for each group, and the average of the 100 replicates was reported.

## 3. Results

### 3.1. Simulated Data

We first simulated one set of pleiotropic traits with 10 shared QTNs and *h*_2_ = 0.5. Their positions and effect sizes are listed in Table 1. Then, pleiotropic variants were explored using both a single-trait model and a PCA-based multiple-trait model. The −log(*p*) and effect standard error (Se Eff) for each QTN are shown in Table 1. Compared with single-trait GWAS, PCA-based multiple-trait GWAS identified additional QTNs. For example, the −log(*p*) of the chr1:132347489 locus in PCA-based GWAS was 6.16, and the corresponding values in the two single-trait GWASs was 4.57 and 5.85. If the significant threshold was *p* < 10^−6^, this locus could be found using PCA-based GWAS, rather than single-trait GWAS.

**Figure 2:**
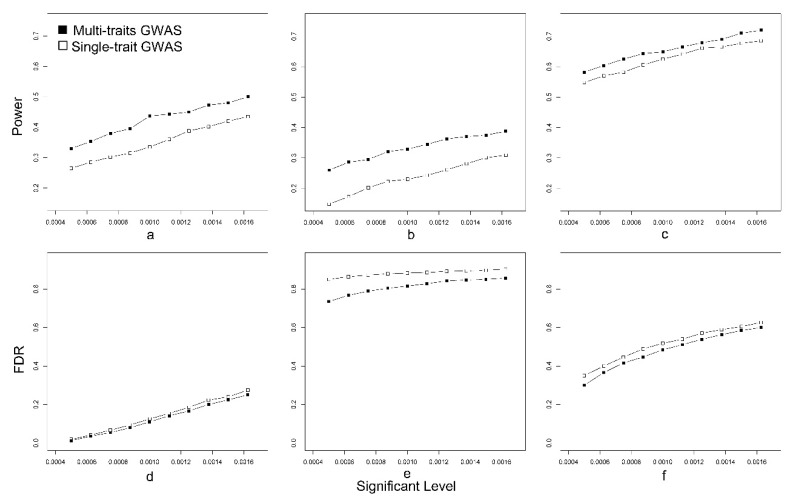
Comparison of power and false discovery rate (FDR) between multiple-trait GWAS and single-trait GWAS. We simulated three situations including medium heritability (**a**,**d**), low heritability (**b**,**e**), and environmental correlation (**c**,**f**). (**a**–**c**) Power under different significant levels. (**d**–**f**) FDR under different significant levels.

To facilitate the comparison of the two association strategies, we compared the power and FDR between them in three situations: Medium heritability (*h*_2_ = 0.5), low heritability (*h*_2_ = 0.05), and environmental correlation (*h*_2_ = 0.5, *r_e_* = 0.25). Table 2 shows phenotypic variance and heritability explained by each principal component (PC) in each scenario. The first dimension (PC1) explained more heritability (*h*_2_ = 0.534, 0.052, and 0.580) compared with the second dimension (*h*_2_ = 0.271, 0.035, and 0.130). As shown in Figure 2a, for medium heritability traits, the power of detection of pleiotropic QTNs in PCA-based GWAS was higher than in single-trait GWAS under different significance thresholds. Additionally, the FDR in multiple-trait GWAS was lower than that in single-trait GWAS (Figure 2d). As expected, the power and FDR decreased with the threshold level becoming stringent. For low heritability traits and environmental correlation traits, we obtained similar results (Figure 2b–f). Overall, PCA-based multiple-trait GWAS outperformed single-trait GWAS in the detection of pleiotropic QTNs.

**Figure 3:**
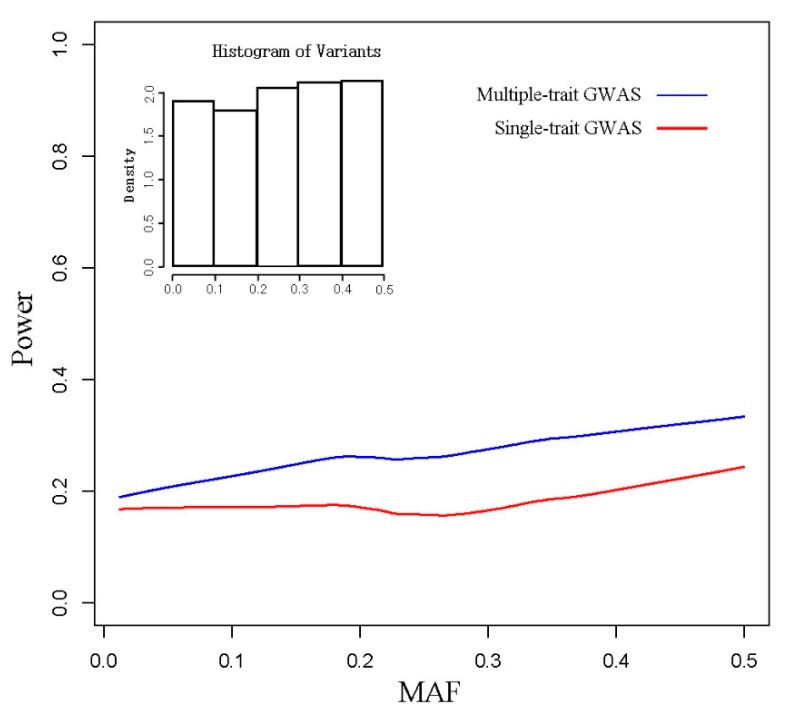
Comparison of detection power between multiple-trait GWAS and single-trait GWAS in different minor allele frequencies. MAF: minor allele frequency. Upper left figure reveals a histogram of the minor allele frequency in the simulated data.

For further investigation, we compared the performance of the two models for different minor allele frequencies (MAFs). In each set of simulations, we first randomly simulated pairwise traits by the pleiotropic QTNs regardless of MAF, and then set a significance threshold of the GWAS results (top 0.04% of the total tested SNPs) to define significant SNPs. The power for each SNP was defined as whether there were significant SNPs harbored by this SNP (1 for harbored, 0 for not harbored). Lastly, based on the power and MAF for each QTN, we fitted trendlines for the two strategies (Figure 3). Overall, PCA-based GWAS outperformed single-trait GWAS. When the MAF of pleiotropic QTNs was less than 0.2, the power difference between them decreased with the reduction of MAF, and when the MAF was greater than 0.2, the differences were maximized and sustained. Since it is hard to define the FDR for each SNP, the relationship between FDR and MAF was not calculated.

**Figure 4:**
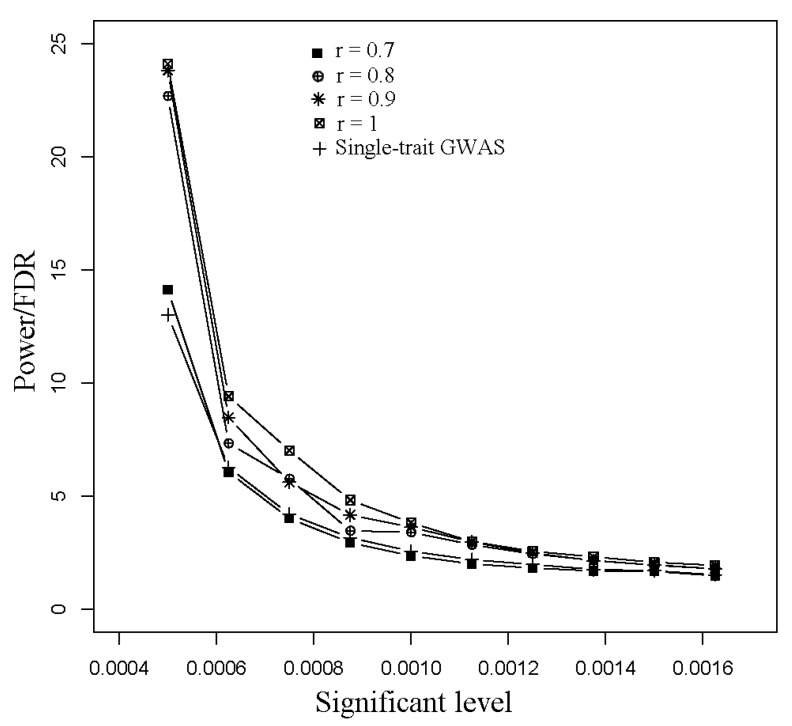
Comparison of power/ False Discover Rate (FDR) in different levels of linkage disequilibrium in the colocalizing effect model.

In the colocalizing effect model, to prove Equation (21), we explored the relationship between the capacity of QTL mapping and linkage disequilibrium (LD) of pleiotropic QTNs. Because the value of power/FDR reflects the statistical power of the GWAS model, we found that the capacity of detection was reduced with decreasing LD of pleiotropic QTNs (Figure 4). For pleiotropic QTNs with *r* = 0.7, PCA-based GWAS had a similar power/FDR to single-trait GWAS.

### 3.2. Real Data

Three meat cut traits, clod weight (CW), fore shank weight (FSW), and heel muscle shank weight (HMSW), are found in presoma muscles and reflect presoma development in cattle. The heritabilities of the three traits ranged from 0.56 to 0.62, and all three traits had a high phenotypic correlation from 0.76 to 0.82, and genetic correlation from 0.90 to 0.94. The details of the descriptive statistics of the three traits are shown in Table 3.

**Figure 5:**
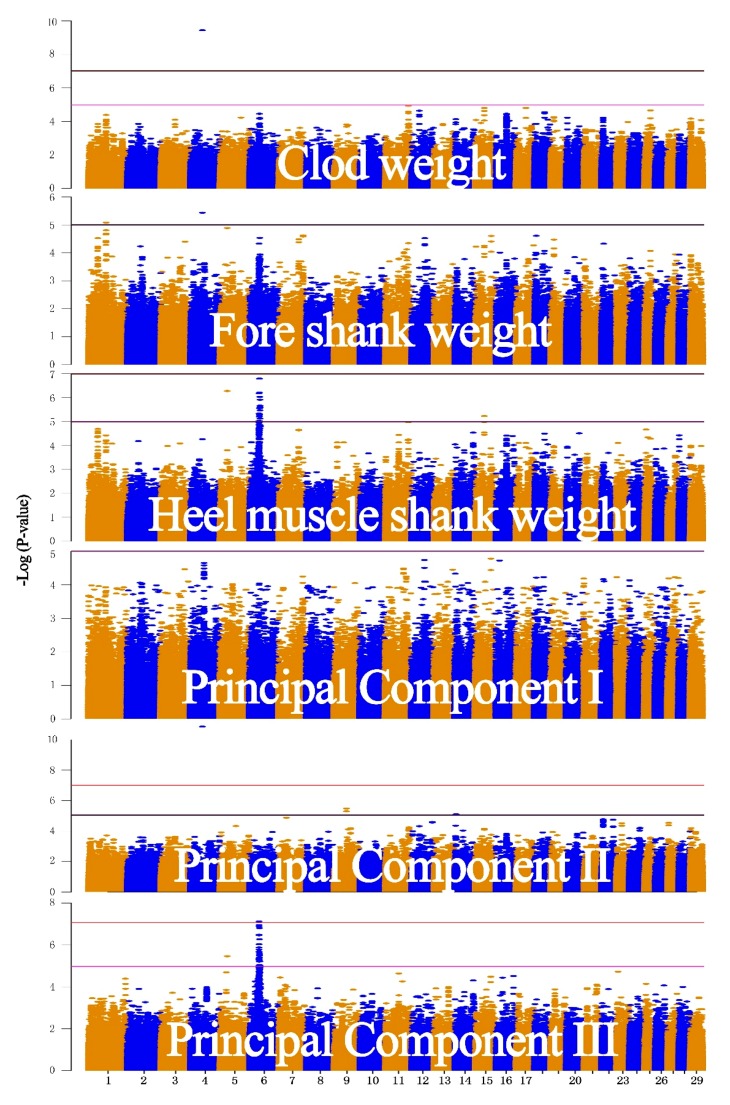
Manhattan plot of the association study results of real cattle data. The three phenotypes are clod weight (CW), fore shank weight (FSW), and heel muscle shank weight (HMSW). The significant level is 10^−7^, represented by the red line, and the suggestive significant level is 10^−5^, represented by the pink line.

GWAS analyses for the three traits were conducted using the single-trait GWAS and PCA-based multiple-trait GWAS strategies (Figure 5). The genome-wide significance threshold and suggestive significance threshold were set at 10^−7^ and 10^−5^, respectively. For CW, only one significant SNP (rs134464739, *p* = 3.64 × 10^−10^) was detected on chromosome 4, and no SNPs exceeded the suggestive significance threshold. For FSW, two significant SNPs (rs134464739 and rs134385681, *p* >10^−5^), one of which was also identified in CW, were detected on chromosomes 1 and 4, respectively. For HMSW, a total of 24 significant SNPs were found (10^−7^ > *p* >10^−5^) on chromosomes 5, 6, and 15. In an approximately 3.5 Mb region (chr6:38550000-42180000), 22 SNPs were associated with the HMSW phenotype, and the most significant SNP was rs137121021, with a *p*-Value of 1.6 × 10^−7^.

In the PCA-based GWAS analysis, the three pseudo traits were combined as new phenotypes (*p*1, *p*2, and *p*3), which explained 86.0%, 8.2%, and 5.8% of the total variance, respectively (Table S1). For the *p*1 GWAS analysis, no significant SNPs were identified. For the *p*2 GWAS analysis, the most significant SNP (rs134464739, *p* = 1.39 × 10^−11^) was also found in CW- and FSW-GWASs. Another four associated SNPs, which exceeded the suggestive significance threshold, were located on chromosomes 9 and 14. For the *p*3 GWAS, in the region (chr6:38550000-42180000) where the HMSW trait was associated with 22 SNPs, a total of 31 significant SNPs were found. Another significant SNP, rs134637644 (3.42 × 10^−6^), on chromosome 5 was also detected by HMSW. Table S2 lists all significant SNPs identified using both methods.

## 4. Discussion

The conception of PCA-based QTL mapping was first introduced by Weller in 1996 [13], in which they found canonical variables can represent original traits effectively. Later on, Mangin et al. (1998) [24] proved that multi-trait analysis was more powerful than single-trait analysis for detecting pleiotropic QTL in QTL mapping analysis. In 2008, Lambertus et al. incorporated heritability parameters into a PCA model, which is a powerful association test model. In 2014, Hugues et al. [15] proposed a combined PCA association model that provides greater flexibility and robustness than other PCA methods. In terms of the power of detecting causal SNPs, most multivariate methods, including the PCA-based method, had similar statistical power [25]. In this study, we evaluated potential improvements to this approach using a broad set of data, both synthetic and real. Theoretically, we derived the relationship between multiple-trait GWAS and single-trait GWAS in two pleiotropy models, as shown in Equations (17) and (21). In Equation (17), we assumed *β*_1_ ≈ *β*_2_ and $r_{y1,y2}>0.7$, resulting in $\hat{\beta^{*}}$ being larger than $\hat{\beta_{1}}$ and $\hat{\beta_{2}}$ (Figure S1). We admitted that a simplified general linear model (GLM) might have bias in comparison with a mixed linear model (MLM), and in Equation (16) there should be cov(*y*_1_, *y*_2_) → *h*_2_ instead of cov(*y*_1_, *y*_2_) → 1 when the environmental correlation equals 0. However, GLM is approximately equivalent to MLM when analyzing unrelated individuals, and traits with genetic correlation show high phenotypic correlation, indicating that the environmental correlation contributes more. In a pleiotropic trait simulation involving medium heritability, low heritability, and environmental correlation, each pairwise trait shared 10 common QTNs that followed a gamma distribution. We found that multiple-trait GWAS outperformed single-trait GWAS in all three situations, which provides some clues that this approach can be applied to a range of pleiotropic traits. In livestock, detection of pleiotropic QTNs has facilitated the biological understanding of commercial traits, particularly in highly related traits, such as birth weight and weaning weight, as well as milk fat yield and milk protein yield. Additionally, due to taxonomic and binary traits in practical breeding programs, we should further optimize the PCA-based multiple-trait model to combine quantitative traits, taxonomic traits, and binary traits.

For the minor allele frequency (MAF), our results indicated that PCA-based GWAS has significant advantages in pleiotropic QTNs detection when the MAF of QTN is greater than 0.2, while the power improvement gradually reduced when the MAF was less than 0.2. Specifically, for uncommon and rare alleles, the PCA-based strategy had little advantage over the single-trait strategy. In the colocalizing effect model, the estimated effect of a pseudo trait is proportional to the level of linkage disequilibrium (LD) (Equation (21)), and the simulation data supported this view (Figure 4). Under the condition that two traits shared pleiotropic QTNs with *r* > 0.7, PCA-based multiple-trait GWAS was more powerful than single-trait GWAS in detecting QTL regions (Figure 4). Assuming that trait 1 had pleiotropic QTNs with trait 2, it was hard to map to this region using single-trait GWAS because of the low LD between the causal variants and genotyped SNP in the beadchip array. However, when there was a high LD between trait 2’s causal variant and the nearby SNP genotyped, this region could potentially be detected in the PCA-based multiple-trait GWAS method after the addition of trait 2.

On the real data, we detected 46 SNPs that were significantly associated with the three traits (Tables S2 and S3). A total of 15 significant SNPs was identified both in single-trait GWAS and multiple-trait GWAS. There were 22 SNPs found only in multiple-trait GWAS, and 9 SNPs found only in single-trait GWAS. Among them, 12 and 18 genes were annotated in multiple-trait GWAS and single-trait GWAS, respectively, which are growth-related genes or muscle development-related genes, such as *NCAPG* [19,26], *LAP3* [27,28], *KCNIP4* [29], and *LCORL* [26,30]. In contrast, six additional genes were found in single-trait GWAS, including *FBXO45*, *SLIT2*, *SMCO1*, *TCTEX1D2*, *UBXN7*, and *WDR53*, which had not been previously reported in growth-associated studies. Only one additional gene, *MCHR2*, was identified in multiple-trait GWAS. Although single-trait GWAS has annotated more genes, it’s result may not be reliable. For example, rs134385681 is a prominent SNP found only in FSW-GWAS which is located in a gene-enriched region, so is likely to be a false positive based on gene annotation. However, *MCHR2* has been reported to be associated with human obesity [31] and a cattle growth trait [32], making it a plausible candidate pleiotropic gene that controls presoma traits.

## 5. Conclusions

In this study, a PCA-based multiple-trait GWAS model proved to be effective in exploring pleiotropic QTNs in theory and practice. Using this method, we found a plausible candidate gene, *MCHR2*, which is associated with presoma muscle development in cattle.

## Figures and Tables

**Table 1:** Positions, effects, and *p*-Values of ten quantitative trait nucleotides (QTNs) based on simulated data without environmental correlation.

Chr ^a^	Pos (bp)	Trait 1 eff	Trait 2 eff	Single-Trait GWAS				Multiple-Trait GWAS	
				−log(*p*) *t*_1_	se eff	−log(*p*) *t*_2_	se eff	−log(*p*) mt	se eff
1	5167453	1.18	1.66	3.63	0.06	1.87	0.09	3.19	0.01
1	126001364	1.34	1.93	4.38	0.03	3.45	0.04	4.65	0.01
1	128776905	1.83	2.51	1.13	0.13	1.17	0.18	1.33	0.03
1	132347489	1.21	1.91	4.57	0.13	5.85	0.18	6.16	0.03
1	135921964	0.89	1.43	1.73	0.06	4.70	0.08	3.53	0.01
4	28841329	0.93	1.47	1.10	0.04	3.68	0.05	2.54	0.01
4	65810279	1.82	2.38	5.24	0.11	5.22	0.16	6.24	0.02
4	80902019	3.41	5.71	17.55	0.06	30.18	0.08	28.08	0.01
4	115266053	2.20	3.94	10.05	0.06	16.65	0.08	15.70	0.01
5	6270944	0.84	0.94	2.48	0.04	0.87	0.05	1.87	0.01

Note: ^a^ One of the simulated data results. Pleiotropic traits were simulated based on 10 QTNs. If the significant threshold was a *p*-Value < 10^−6^, only two QTNs (chr4: 80902019 and chr4: 115266053) could be identified based on single-trait GWAS results. Meanwhile, four QTNs (chr1: 132347489, chr4: 65810279, chr4: 80902019, and chr4: 115266053) could be identified based on PCA-based GWAS results. Shaded QTNs are causal variants only found in PCA-based GWAS. GWAS, Genome-Wide Association Study. Chr, Chromosome. Pos, Position. Eff, effective. Se eff, Standard error of estimated effects.

**Table 2:** Phenotypic variance and heritability explained by each principle component.

Scenario	Heritability	Environmental Correlation	PC1		PC2	
			Phenotypic Variance (SD ^a^)	Heritability Explained (SD)	Phenotypic Variance (SD)	Heritability Explained (SD)
1	0.5	0	75.98 (25.12)	0.534 (0.04)	14.96 (4.34)	0.271 (0.03)
2	0.05	0	56.78 (17.22)	0.052 (0.01)	39.81 (10.23)	0.035 (0.01)
3	0.5	0.25	89.12 (30.09)	0.580 (0.04)	9.80 (2.11)	0.130 (0.07)

Note: ^a^ SD: Standard Deviation.

**Table 3:** Statistical summary and genetic parameters of three phenotypes.

Trait	Number of Samples	Mean (Kg) (SD)	Heritability	CW	FSW	HMSW
Clod weight (CW)	1111	5.06 (0.88)	0.57	1	0.82 ^a^	0.79
Fore shank weight (FSW)	1111	17.03 (3.15)	0.56	0.90 ^b^	1	0.76
Heel muscle shank weight (HMSW)	1111	1.07 (0.19)	0.62	0.93	0.94	1

Note: ^a^ phenotype correlation. ^b^ genetic correlation.

## Supplementary Materials

The following are available online at http://www.mdpi.com/2076-2615/8/12/239/s1, Figure S1: Summation of eigenvectors at different correlation levels based on summated data. Table S1: Component matrix and total variance explained by each principal component. Table S2: Significant SNPs and candidate genes associated with three traits in single-trait GWAS. Table S3: Significant SNPs and candidate genes associated with three traits in PCA-based multiple-trait GWAS.
