# Supplementary material for: PCA-Based Multiple-Trait GWAS Analysis: A Powerful Model for Exploring Pleiotropy
Source: Animals (Basel). 2018 Dec 17;8(12):239. doi: 10.3390/ani8120239 (PMC6316348; doi:10.3390/ani8120239)
Supplement: Supplementary file 1 [file animals-08-00239-s001.pdf]

animals

MDPI

# Supplementary files: PCA-Based Multiple-Trait GWAS Analysis: A Powerful Model for Exploring Pleiotropy

**Wengang Zhang [1,†], Xue Gao [1,†], Xinping Shi [1,2], Bo Zhu [1], Zezhao Wang [1], Huijiang Gao [1], Lingyang Xu [1], Lupei Zhang [1], Junya Li [1,*] and Yan Chen [1,*]**

1. Cattle Genetics and Breeding Group, Institute of Animal Science (IAS), Chinese Academy of Agricultural Sciences (CAAS), Beijing 100193, China; zhangwengang_19@sina.com (W.Z.); gaoxue76@126.com (X.G.); sxp18811727129@163.com (X.S.); zhubo525@126.com (B.Z.); wangzezhao1@163.com (Z.W.); gaohj111@sina.com (H.G.); xulingyang@caas.cn (L.X.); zhanglupei@caas.cn (L.Z.)
2. College of Animal Science and Technology, Hebei Agricultural University, Baoding 071000, China

* Correspondence: lijunya@caas.cn (J.L.); chenyan0204@163.com (Y.C.); Tel.: +86-138-1156-8766 (J.L.); +86-134-3967-4745 (Y.C.)

† These authors contributed equally to this work.

Received: 30 August 2018; Accepted: 28 November 2018; Published: 30 November 2018

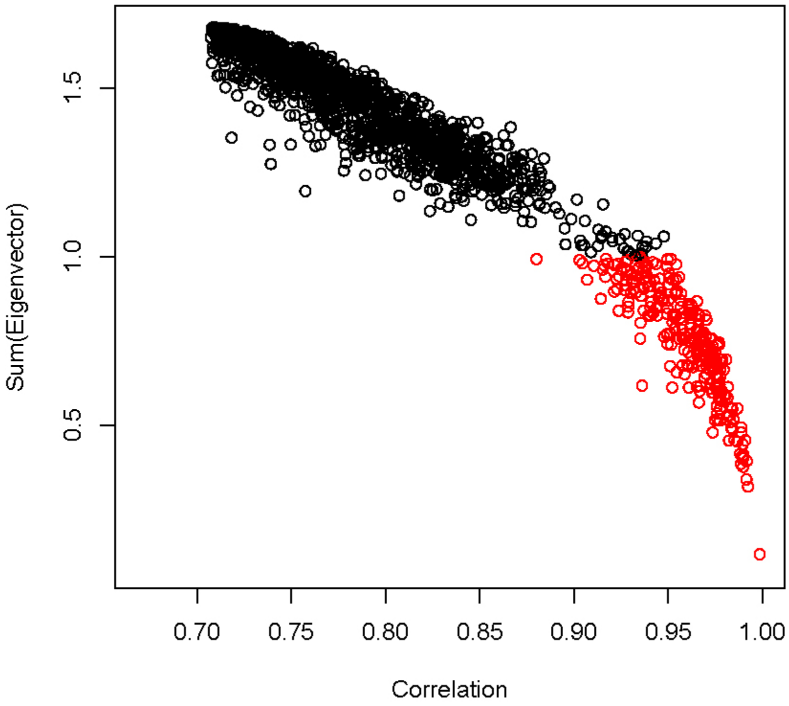


**Figure S1.** Summation of eigenvectors at different correlation levels based on summated data. Red plots represent that the summation of the eigenvector is lower than 1.

**Table S1.** Component matrix and total variance explained by each principal component.

| Trait | Principal Component | | |
|---|---|---|---|
| | I | II | III |
| Clod weight | 0.915 | 0.395 | 0.084 |
| Heel muscle shank weight | 0.928 | –0.279 | 0.247 |
| Fore shank weight | 0.939 | –0.109 | –0.326 |
| Total variance explained | 86.0% | 8.2% | 5.8% |

**Table S2.** Significant SNPs and candidate genes associated with three traits in single-trait GWAS.

| BTA | SNP name | Position | *p*-Value | Annotated gene | Trait [1] |
|---|---|---|---|---|---|
| 6 | BovineHD0600010958 | 40003884 | $1.60\times 10^{-7}$ | | HMS |
| 6 | BovineHD0600010960 | 40006739 | $1.60\times 10^{-7}$ | | HMS |
| 5 | BovineHD0500008693 | 29684213 | $5.27\times 10^{-7}$ | LARP4 FAM186A | HMS |
| 6 | BovineHD0600010961 | 40007899 | $6.15\times 10^{-7}$ | | HMS |
| 6 | BovineHD4100004580 | 38852093 | $6.56\times 10^{-7}$ | DCAF16 NCAPG LCORL | HMS |
| 6 | BovineHD0600011236 | 41498235 | $9.31\times 10^{-7}$ | SLIT2 | HMS |
| 6 | BTA-75902-no-rs | 42155077 | $1.22\times 10^{-6}$ | KCNIP4 | HMS |
| 6 | BovineHD0600011439 | 42167429 | $2.17\times 10^{-6}$ | KCNIP4 | HMS |
| 6 | BovineHD0600011438 | 42162209 | $2.21\times 10^{-6}$ | KCNIP4 | HMS |
| 6 | BovineHD0600011442 | 42175002 | $2.49\times 10^{-6}$ | KCNIP4 | HMS |
| 6 | BovineHD0600011441 | 42169746 | $2.64\times 10^{-6}$ | KCNIP4 | HMS |
| 6 | BovineHD0600010952 | 39990876 | $2.78\times 10^{-6}$ | | HMS |
| 6 | BovineHD0600011435 | 42154344 | $3.37\times 10^{-6}$ | KCNIP4 | HMS |
| 6 | BovineHD0600010660 | 38549807 | $4.61\times 10^{-6}$ | LAP3 MED28 FAM184B | HMS |
| 6 | BovineHD0600010950 | 39988294 | $4.83\times 10^{-6}$ | | HMS |
| 6 | BovineHD0600010951 | 39989868 | $5.06\times 10^{-6}$ | | HMS |
| 6 | BovineHD0600010956 | 39997880 | $5.26\times 10^{-6}$ | | HMS |
| 15 | BovineHD1500010212 | 37272475 | $5.90\times 10^{-6}$ | | HMS |
| 6 | BovineHD0600010953 | 39991818 | $6.08\times 10^{-6}$ | | HMS |
| 6 | BovineHD0600010827 | 39372172 | $6.27\times 10^{-6}$ | | HMS |
| 6 | BovineHD0600011437 | 42158231 | $7.45\times 10^{-6}$ | KCNIP4 | HMS |
| 6 | BovineHD0600011436 | 42156523 | $7.47\times 10^{-6}$ | KCNIP4 | HMS |
| 6 | Hapmap26308-BTC-057761 | 38576012 | $9.41\times 10^{-6}$ | LAP3 MED28 FAM184B | HMS |
| 6 | BovineHD0600011226 | 41457949 | $9.75\times 10^{-6}$ | SLIT2 | HMS |
| 4 | BovineHD0400013305 | 48381566 | $3.64\times 10^{-10}$ | PIK3CG PRKAR2B | CW |
| 4 | BovineHD0400013305 | 48381566 | $3.64\times 10^{-6}$ | PIK3CG PRKAR2B | FS |
| 1 | BovineHD0100020542 | 71613561 | $8.25\times 10^{-6}$ | TCTEX1D2 UBXN7 SMCO1 WDR53 FBXO45 | FS |

Note: [1] HMS, heel muscle weight; CW, clod weight. BTA, *Bos taurus* autosome. SNP, single nucleotide polymorphism.

**Table S3.** Significant SNPs and candidate genes associated with three traits in PCA-based multiple-trait GWAS.

| BTA | SNP name | Position | *p*-Value | Gene | PCA |
|---|---|---|---|---|---|
| 4 | BovineHD0400013305 | 48381566 | $1.39\times 10^{-11}$ | | PC2 |
| 9 | BovineHD0900013932 | 50518738 | $3.28\times 10^{-6}$ | MCHR2 | PC2 |
| 9 | BovineHD0900013931 | 50517026 | $5.06\times 10^{-6}$ | MCHR2 | PC2 |
| 14 | BovineHD4100010732 | 5888713 | $7.85\times 10^{-6}$ | | PC2 |
| 14 | BovineHD4100010731 | 5887202 | $8.17\times 10^{-6}$ | | PC2 |
| 6 | BovineHD4100004565 | 38471732 | $7.55\times 10^{-8}$ | LAP3 | PC3 |
| 6 | BovineHD4100004566 | 38477781 | $1.12\times 10^{-7}$ | LAP3 | PC3 |
| 6 | Hapmap30134-BTC-034283 | 38464203 | $1.29\times 10^{-7}$ | | PC3 |
| 6 | BovineHD0600010958 | 40003884 | $1.56\times 10^{-7}$ | | PC3 |
| 6 | BovineHD0600010960 | 40006739 | $1.56\times 10^{-7}$ | | PC3 |
| 6 | BovineHD0600010647 | 38469930 | $3.23\times 10^{-7}$ | | PC3 |
| 6 | BovineHD0600010660 | 38549807 | $3.43\times 10^{-7}$ | | PC3 |
| 6 | BovineHD0600010961 | 40007899 | $5.34\times 10^{-7}$ | | PC3 |
| 6 | BovineHD0600010666 | 38574125 | $9.28\times 10^{-7}$ | LAP3 MED28 FAM184B | PC3 |
| 6 | BovineHD0600010669 | 38583783 | $9.28\times 10^{-7}$ | LAP3 MED28 FAM184B | PC3 |
| 6 | BovineHD0600010676 | 38595246 | $1.32\times 10^{-6}$ | LAP3 MED28 FAM184B | PC3 |
| 6 | BovineHD0600010668 | 38581382 | $1.66\times 10^{-6}$ | LAP3 MED28 FAM184B | PC3 |
| 6 | BovineHD0600010648 | 38471181 | $2.07\times 10^{-6}$ | LAP3 MED28 FAM184B | PC3 |
| 6 | BovineHD0600010649 | 38474338 | $2.15\times 10^{-6}$ | | PC3 |
| 6 | BovineHD0600010952 | 39990876 | $2.50\times 10^{-6}$ | | PC3 |
| 6 | BovineHD0600010950 | 39988294 | $2.88\times 10^{-6}$ | | PC3 |

| | | | | | |
|---|---|---|---|---|---|
| 6 | BovineHD0600010951 | 39989868 | $3.10\times 10^{-6}$ | | PC3 |
| 6 | BovineHD0600010953 | 39991818 | $3.22\times 10^{-6}$ | | PC3 |
| 5 | BovineHD0500008693 | 29684213 | $3.42\times 10^{-6}$ | LARP4 FAM186A | PC3 |
| 6 | BovineHD0600010956 | 39997880 | $3.98\times 10^{-6}$ | | PC3 |
| 6 | BovineHD4100004570 | 38494379 | $4.19\times 10^{-6}$ | | PC3 |
| 6 | BovineHD0600010653 | 38495361 | $4.53\times 10^{-6}$ | LAP3 MED28 FAM184B | PC3 |
| 6 | BovineHD0600010665 | 38571977 | $4.54\times 10^{-6}$ | LAP3 MED28 FAM184B | PC3 |
| 6 | BovineHD0600010686 | 38618402 | $4.85\times 10^{-6}$ | LAP3 MED28 FAM184B | PC3 |
| 6 | BovineHD4100004580 | 38852093 | $5.04\times 10^{-6}$ | NCAPG LCORL DCAF16 | PC3 |
| 6 | BovineHD4100004568 | 38487018 | $5.29\times 10^{-6}$ | LAP3 | PC3 |
| 6 | BTA-75902-no-rs | 42155077 | $6.02\times 10^{-6}$ | KCNIP4 | PC3 |
| 6 | BovineHD0600010824 | 39356766 | $6.26\times 10^{-6}$ | | PC3 |
| 6 | BovineHD0600010957 | 40000025 | $9.01\times 10^{-6}$ | | PC3 |
| 6 | BovineHD0600011439 | 42167429 | $9.57\times 10^{-6}$ | KCNIP4 | PC3 |
| 6 | BovineHD0600011438 | 42162209 | $9.75\times 10^{-6}$ | KCNIP4 | PC3 |
| 6 | BovineHD0600010684 | 38614787 | $9.76\times 10^{-6}$ | LAP3 MED28 FAM184B | PC3 |

Note: BTA, *Bos taurus* autosome. SNP, single nucleotide polymorphism. PCA, principal component analysis.
